# Too Numerous to Enumerate in Half Page

**Published:** 1886-09

**Authors:** 


### EDITORIAL NOTES.

DR. FRANK HASTINGS HAMILTON, who died recently, is the last of the great trio of American surgeon-physicians, whose life and labors distinguished the present era—Gross, Flint and Hamilton.

YELLOW FEVER was officially declared in New Orleans September 1st. State Health Officer Swearingen, of Texas, went over to investigate, and, we presume, quaratine against New Orleans will be immediately declared.

TRANSPLANTATION OF A RABBIT'S EYE.–The *New England Medical Monthly* says that Dr. N. O. Bradford, of Boston, has a second time successfully performed the operation of transplanting a rabbit's eye into the orbit of a man. This makes in all, as far as reported, six cases where the attempt has been made, and Dr. Bradford—an American surgeon—is the only one who has succeeded, and he has performed the very extraordinary operation successfully twice. Altho' vision was not expected in the transplanted eye, says the *New England Medical Monthly*, the patient obtained a very satisfactory artificial eye.

THE TEXAS COURIER RECORD OF MEDICINE, our sprightly and enterprising neighbor, has removed to Dallas. We are much pleased to note a great improvement in the typographical execution of the journal, and in its general appearance and get up. We wish our friends, the two big B's every success.

---

SCIENTIFIC INTOLERANCE.—"How can there be such a question as Science versus Religion? Both are supposed to be expressions of great truths, and though we may not at first sight recognize the harmony of separate presentation, yet if they be not falsities, each is compatible, the one with the other. But if any man attempts an appeal to bigotry and prejudice when a new scientific theory is broached, and endeavors to forestall a candid examination by denouncing it as not in harmony with any formulated creed, whether of revealed religion or practical knowledge, he is reviving the methods of the old inquisition, which gave to Galileo the choice between recantation and the stake. Creeds are but the work of men, and in the light of further knowledge even the best may be found defective.

It is always easier to create a prejudice against an argument by branding it heterodox, than carefully to examine into its merits or demerits. But in the name of all that is consistent, why should political or religious questions enter into the consideration of strictly scientific subjects? If the theories be true they cannot but harmonize with religious truth, and if they be false that must appear upon an examination. The aim of professional societies is supposed to be the elimination of extraneous matter, and the bringing to light of scientific facts, no matter what particular creed or party or faction may fancy itself prejudiced thereby. What matters it, what political party or religious sect a man may belong to if he reveals to us new teachings of science? Is there not even one place where men of all creeds can meet on common ground, and intelligently discuss scientific and professional questions without engaging in personal, political, or religious polemics? *This branding of men as dangerous creatures because they do not subscribe to some other man's dogma, is unworthy those who are searching after truth.* [Ital ours. Ed.] What man possesses a creed which all other classes of men will pronounce orthodox? It is every man's privilege to stand up stoutly for his political or religious belief when it is attacked, but it is despicable to appeal to sectarian or party prejudice to prevent the consideration of scientific theories in a professional meeting."—*Editorial, Independent Practitioner.*

The above expresses our ideas so fully on certain points, now engaging the attention of our own profession, that we produce it almost entire.

We hold that science and religion are not antagonistic, but on the contrary, prove each other ; and we believe that the apparent inconsistencies—for they are only imaginary—will be cleared up, and reconciled, in the light of better knowledge—as man "advances in intellectual development."

But, we repeat what we said on a former occasion, the discussion of religious beliefs, is "out of place before a medical association."

---

"A NOTABLE ADDRESS."—Dr. Merryman says Dr. Billings' address was not—able in one respect; it was very weak. Malaria did it.

---

SACCHARINE—VERY !—It is claimed that a substance has been discovered, a principle of coal tar, which is two hundred and thirty seven times sweeter than cane sugar.

Our Dr. Merryman says he is acquainted with something just two hundred and thirty seven thousand times sweeter than saccharine. It is also a "principal"—and her name is *Cather*—ine !

---

A MEMBER of Congress, from the West, received the following from one of his constituents : "Dear Sir, my children have been afflicted with scabs all winter, and the medicine given them by the doctor here does not seem to do them any good. I see by the papers that there are some very fine doctors in Washington connected with the Government, and if it does not cost too much I wish you would ask them what is good for the scabs, and write me by return mail. "—*Exchange.*

We learn by special from Washington that the Congressman referred the case to Dr. JNO. S. BILLINGS, U. S. A.—as being the *finest* of the " fine doctors connected with the Government," and an authority on " scabs." This is in keeping with the eternal fitness of things. Billings—the "exception to the rule" having been raised in the West, and having carried all the science out of that section when he removed to Washington, is, in a measure, responsible for this boy's case. (See " Malarial Map.")—The writer, when he says, "I see by the papers," evidently had read that British address—how else would he know that the "fine doctors are connected with the Government?"

---

MR. E. T. COOK, brother of Dr. F. T. Cook of Taylor, died at his home in Williamson Co., August 4, of typhoid fever.

---

DR. W. F. SHARPE of Milano is a candidate for representative in the next legislature.

---

DR. T. J. WILSON, assistant physician at the Insane Asylum at Austin, has resigned, and will return to Sherman, his old home.

---

Dr. T. J. BENNETT, of Austin, has received the appointment of 1st Assistant Physician to the State Insane Asylum, vice Wilson resigned.

---

A WHISPER TO OUR FRIENDS.—We know that times are hard and money scarce in the country. Although our terms are $2, payable in advance, and notwithstanding it takes money to run the JOURNAL, we do not wish to be understood as *pressing* our subscribers; certainly not as making it an alternative to pay or stop. While we will be very thankful for all favors in the way of advance subscriptions, and we ask those who can, conveniently, do so, to remit, we will *cheerfully* extent time to all who desire it—who desire to continue the JOURNAL, but are not prepared just now to pay for it. Drop us a card and a memorandum will be made opposite your name, to await your convenience.

---

Referring to the excellent article on "Gunshot Wounds of the Spinal Column," the last paper (but one) written by the lamented Dr. Burt,—and which is published in this number of the JOURNAL, it is much to be regretted that he left no key to the cuts representing the injured vertebræ;—the cuts were ordered, but only arrived after his death. This detracts greatly from the value of the contribution.

---

IT BEATS THE DECK!—*The Texas Courier Record of Medicine* and DANIEL'S TEXAS MEDICAL JOURNAL club for $3.00! Address either office. Time limited.

